# Cingulum-Callosal white-matter microstructure associated with emotional dysregulation in children: A diffusion tensor imaging study

**DOI:** 10.1016/j.nicl.2020.102266

**Published:** 2020-04-25

**Authors:** Yuwen Hung, Mai Uchida, Schuyler L. Gaillard, Hilary Woodworth, Caroline Kelberman, James Capella, Kelly Kadlec, Mathias Goncalves, Satrajit Ghosh, Anastasia Yendiki, Xiaoqian J. Chai, Dina R. Hirshfeld-Becker, Susan Whitfield-Gabrieli, John D.E. Gabrieli, Joseph Biederman

**Affiliations:** a Athinoula A. Martinos Imaging Center at the McGovern Institute for Brain Research, Massachusetts Institute of Technology and Harvard University, Cambridge, MA 02139, United States; b Department of Brain and Cognitive Sciences, Cambridge, MIT, MA 02139, United States; c Department of Psychiatry, Harvard Medical School, Boston, MA 02115, United States; d Clinical and Research Program in Pediatric Psychopharmacology and Adult ADHD, Massachusetts General Hospital, Boston, MA 02114, United States; e Child Cognitive Behavioral Therapy Program, Massachusetts General Hospital, Boston, MA 02114, United States; f Department of Radiology, Harvard Medical School, Boston, MA; g Department of Neurology and Neurosurgery, McGill University; h Department of Electrical and Computer Engineering and School of Neuroscience, Virginia Tech, Blacksburg, VA 24061, United States; i Department of Biomedical Sciences, Tufts University School of Medicine, Boston, MA 02111, USA; j Institute for Medical Engineering and Science, MIT, Cambridge, MA 02139, United States

**Keywords:** Emotional dysregulation, Mood disorders, Limbic system, Cingulum, Corpus callosum

## Abstract

Emotional dysregulation symptoms in youth frequently predispose individuals to increased risk for mood disorders and other mental health difficulties. These symptoms are also known as a behavioral risk marker in predicting pediatric mood disorders. The underlying neural mechanism of emotional dysregulation, however, remains unclear. This study **used** the diffusion tensor imaging (DTI) technique to identify anatomically specific **variation** in white-matter microstructure that is associated with pediatric emotional dysregulation severity. Thirty-two children (mean age 9.53 years) with varying levels of emotional dysregulation symptoms were recruited by the Massachusetts General Hospital and underwent the DTI scans at Massachusetts Institute of Technology. Emotional dysregulation severity was measured by the empirically-derived Child Behavior Checklist Emotional Dysregulation Profile that includes the Attention, Aggression, and Anxiety/Depression subscales. Whole-brain voxel-wise regression tests revealed significantly increased radial diffusivity (RD) and decreased fractional anisotropy (FA) in the cingulum-callosal regions linked to greater emotional dysregulation in the children. The results suggest that **microstructural differences** in cingulum-callosal white-matter pathways may manifest as a neurodevelopmental vulnerability for pediatric mood disorders as implicated in the clinical phenotype of pediatric emotional dysregulation. These findings may offer clinically and biologically relevant neural targets for early identification and prevention efforts for pediatric mood disorders.

## Highlights

• Emotional dysregulation is related to differences in cingulum-callosal microstructural integrity.
• Emotional dysregulation is associated with increases in radial diffusivity and decreases in fractional anisotropy in cingulum-callosal bundles.
• Cingulum-callosal bundles may be susceptibility neuromarkers for pediatric mood disorders.

## 1 Introduction

Emotional dysregulation (ED) is a frequently encountered pediatric behavioral and emotional manifestation predictive of subsequent mood disorder. It is characterized by a set of symptoms in which children fail to manage their emotions, resulting in quickness to anger, inability to refocus attention from strong emotions, and low frustration tolerance (Biederman et al., 2012a; Biederman et al., 2012b). Better emotion regulation in youth has been found to be protective against adverse physical and mental health outcomes (Bell and McBride, 2010; National Research Council and Institute of Medicine, 2009), and longitudinal evidence has shown that ED in childhood predicts subsequent onset of mood disorders and suicidality (Biederman et al., 2009; Holtmann et al., 2011). In addition, ED symptoms have been attributed to increases in comorbidity among pediatric psychiatric disorders and are associated with more severe functionally impairing outcomes (Arnold et al., 2011; Biederman et al., 2012a; Biederman et al., 2012b; O'Brien and Frick, 1996). Therefore, emotional dysregulation in childhood is known to represent a prodrome to various adult psychopathologies (Bertocci et al., 2016). Here, we asked whether emotional dysregulation symptom in children can be identified by anatomically specific differences in white-matter microstructure.

Emotion regulation is conceptualized as top-down regulatory processes associated with prefrontal and medial frontal control over bottom-up emotional reactivity associated with limbic structures (Gross, 2013; Ochsner et al., 2002; Ochsner et al., 2004). Therefore, ED symptoms may reflect difficulty in the cortical regulation of emotions. This suggests that the strength of brain connection between the emotion-regulating frontal cortical regions and the emotion-generating limbic subcortical structures may be diminished in children struggling with ED. We hypothesized that abnormal connectional microstructure between the frontal cortical and the subcortical (limbic) systems would be predictive of ED symptom severity in children. Functional neuroimaging evidence concerning pediatric ED suggests abnormalities in amygdala-insula resting-state connectivity (Bebko et al., 2015) and in functional activation of prefrontal regions (Bertocci et al., 2014), the limbic system (Bertocci et al., 2014; Tseng et al., 2016), and fronto-limbic and sensorimotor areas (Portugal et al., 2016)***.*** Existing structural imaging data in youth regarding emotional dysregulation are limited, and prior investigations were restricted to pre-defined white-matter tracts of interest (Bertocci et al., 2016; Versace et al., 2015). One study in youth regarding ED reported that greater cingulum length (and not psychiatric diagnosis) at baseline assessment predicted lesser emotional dysregulation in a 14.2-month longitudinal follow-up as measured by a manic behavior scale (Bertocci et al., 2016). Another study in youth with multiple different psychiatric diagnoses categorized by emotional versus behavioral dysregulation characteristics reported abnormalities of white-matter microstructure (decreased fractional anisotropy and axial diffusivity) in uncinate fasciculus and cingulum tracts associated with the emotional dysregulation (Versace et al., 2015).

This study aligns with the NIH Research Domain Criteria (RDoC) (Insel et al., 2010) (https://www.nimh.nih.gov/research-priorities/rdoc/index.shtml) that expand from the conventional diagnosis-driven to a dimensional-based approach, and aims to improve current understanding of the etiology of pediatric mood disorders. We combined a clinical dimensional approach (a clinical-based ED profile derived from the Child Behavior Checklist (CBCL)) and imaging method (diffusion tensor imaging (DTI)) to characterize neuroanatomical correlates of ED. Within the CBCL, the composite standard T scores combining the Attention, Aggression, and Anxiety/Depression subscales (A-A-A) effectively identify children with various levels of ED symptoms and longitudinally predict subsequent onset of mood disorders and suicidality (Achenbach, 1991; Biederman et al., 2009; Biederman et al., 2012a; Biederman et al., 2012b). This specific profile—CBCL-Emotional Dysregulation (ED) (Achenbach, 1991; Biederman et al., 2009; Biederman et al., 2012a; Biederman et al., 2012b)—has been useful to supplement structured interviews for screening lifetime and current diagnoses of major depressive and bipolar disorders (Carlson and Kelly, 1998; Faraone et al., 2005; Geller et al., 1998; Hazell et al., 1999; Mick et al., 2003; Uchida et al., 2014; Wals et al., 2001). An abnormal CBCL-ED profile (i.e., combined T score of the A-A-*A* > 180) helps identify children with increased susceptibility to developing mood disorders; and a severe ED profile (i.e., T score of A-A-*A* > 210) has been particularly sensitive for screening pediatric bipolar disorder (Faraone et al., 2005; Mick et al., 2003; Uchida et al., 2014). In the current study, we investigated 32 children (mean age 9.53 years) with low to high levels of ED symptoms determined by the CBCL-ED measure, and employed DTI techniques to determine the brain connectional microstructure underlying the clinical dimension of ED. We used a conservative, whole-brain voxelwise search approach to reveal **variations** in white-matter microstructure associated with ED severity. This approach allowed us to identify regional significance and locate brain pathways in major white-matter tracts.

This study is the first investigation attempting to relate a well-validated clinical measurement of emotion dysregulation (CBCL-ED profile) with a whole-brain measure of brain structure, specifically white-matter microstructure. The whole-brain analysis allowed for evidence of regional specificity about any white-matter correlates of ED symptoms (prior studies only examined restricted regions of interest). Identifying neural substrates underlying pediatric ED is of critical clinical importance because such knowledge could be used to provide biologically-relevant neurodevelopmental targets for early detection and prevention of mood disorders, guiding treatment choices and aiding in novel therapeutic approaches for children at-risk (Hasler et al., 2006; Phillips and Frank, 2006). The outcome of this study could improve the understanding of the neural susceptibility for bipolar mood disorders and may contribute to preventive strategies for young cohorts at risk.

## 2 Material and Methods

### 2.1 Participants

**Fig. 1:**
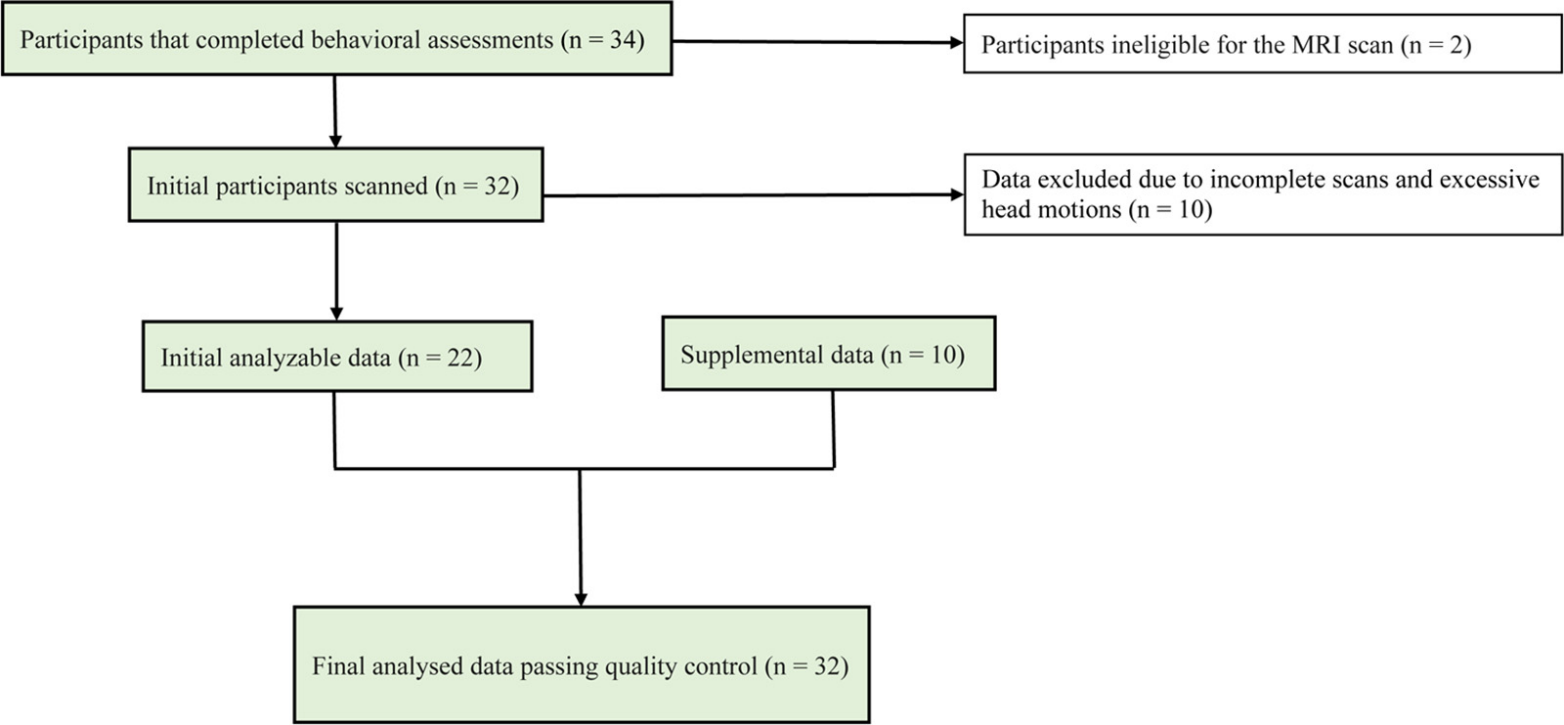
PRISMA Diagram of subject enrollment. Breakdown of participant recruitment, screening procedures, and eligible data for analysis.

Participants were recruited from the community by Massachusetts General Hospital (MGH). Fig. 1 presents the PRISMA flow diagram detailing subject enrollment. The final sample for the study consisted of 32 children (mean age = 9.53, SD = 1.83; 16 boys and 16 girls) with low to high degree of ED difficulties. Children with neuroimaging contraindications, suicidality, psychosis, and risk of harming others were excluded. **The effect of age was controlled in all statistics as a covariate. The original data were supplemented with 10 children from a prior study's control group (****Chai et al., 2016****) who underwent the same psychiatric and neuroimaging assessment procedures using the same scanner**. . The effect of participants from different sources was statistically controlled as a covariate in **all** analyses. As the current study focuses on the dimensional rather than diagnostic approach, we did not exclude children with lifetime history of depression. Seven children had lifetime history of mood disorders, which was statistically controlled for in all analyses. This study was approved by the Institutional Review Boards of MGH and Massachusetts Institute of Technology (MIT). (MGH approval ID: 2014P000439; MIT approval ID: 1,414,006,349). Written informed consent from all parents and assent from all child participants were obtained.

### 2.2 Assessment of Emotional Dysregulation—Child Behavior Checklist (CBCL)

**Fig. 2:**
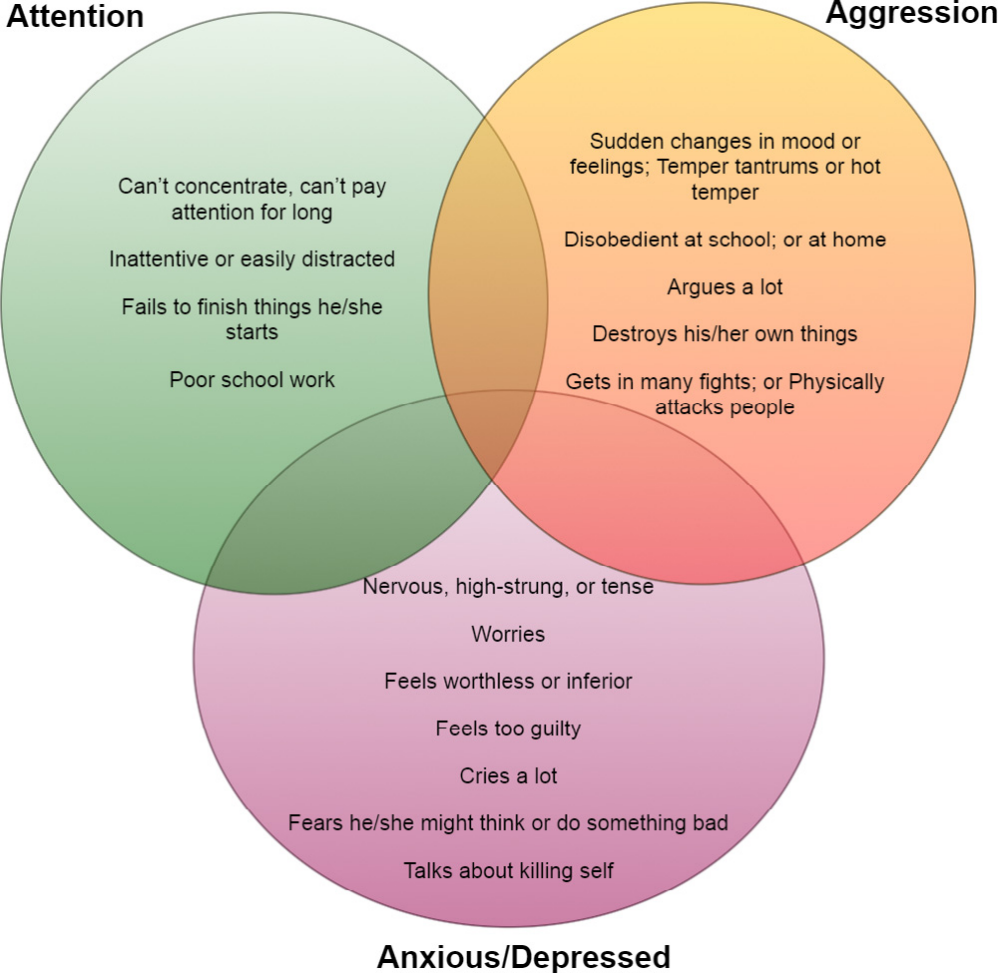
The CBCL-ED profile and T score distribution. The CBCL-ED profile, or AAA score, is a composite standardized T score combining three empirical syndromal subscales of the CBCL: The Anxious/Depressed subscale, the Attention Problems subscale, and the Aggressive Behavior subscale. Higher AAA score indicates greater ED. Abnormal CBCL-ED profile (i.e., AAA score > 180) helps identify children with increased susceptibility to developing mood disorders. Youth's ED profile has longitudinally predicted subsequent onset of mood disorders and suicidality, and severe CBCL-ED score (i.e., AAA ≥ 210) is particularly sensitive for screening for pediatric bipolar disorder. The figure is produced with granted permission by dr. Achenbach.

The CBCL is a widely used clinical tool with empirically derived scales and excellent psychometric properties (Achenbach, 1991; Althoff et al., 2006; Faraone et al., 2005; Hudziak et al., 2005; Hudziak et al., 2005; Mick et al., 2003). It characterizes a child's behavior in the past six months by parent-report, and the data are transformed into dimensional behavioral problem standard scores (Angold et al., 1998). All raw scores were converted to standardized T scores for clinical use based on age and gender by a computerized program. The CBCL-ED profile was derived from a composite score combining the standardized T scores of the three (A-A-A) empirical syndrome scales: The CBCL Anxious/Depressed subscale, Attention Problems subscale, and Aggressive Behavior subscale (Fig. 2). Higher AAA score indicates greater ED severity. The CBCL assigns a minimum T score of 50 to any syndrome scale truncating low-end raw scores that are considered clinically normal, and symptom-free (Achenbach and Rescorla, 2001; Achenbach, 1991). We include participants with symptom-positive ED profiles with ED scores > 150 that are clinically meaningful, and exclude symptom-free participants (ED score = 150). The participants’ ED scores in the study ranged from 151 to 265. The 1991 version of the CBCL for children between 6 and 18 years was completed by the participants’ parents.

### 2.3 Psychiatric Assessment

The Kiddie Schedule for Affective Disorder and Schizophrenia-Epidemiological version (KSADS-E) modules on mood disorders were used to define the presence or absence of major depressive or bipolar disorder (Orvaschel, 1994). The KSADS-E is a semi-structured psychiatric diagnostic interview designed for use in clinical and epidemiologic research to obtain past and current history of psychiatric disorders for children ages 6 to 17 years.

### 2.4 Magnetic Resonance and Diffusion Tensor Imaging

All participants underwent MRI scanning, including T1-weighted whole-head anatomical and diffusion-weighted imaging scans in the same session at the Martinos Imaging Center at the McGovern Institute for Brain Research at MIT. Imaging data were acquired on a 3 Tesla Siemens Trio scanner using a 32-channel head coil. T1 MPRAGE sequence parameters included 1.1 × 1.1 mm2 in-plane resolution, 1.0 mm slice thickness, field of view (FOV) = 247 × 247 mm2, matrix = 220 × 220, 176 slices, four-echo sequence with TE = 1.57 ms, 3.33 ms, 5.09 ms, and 6.85 ms, and TR = 2.53 s. Prospective acquisition correction was used to mitigate artifacts due to head motion. The diffusion-weighted scan sequence included 1 non-diffusion weighted reference volume (*b* = 0) and 30 diffusion directions (*b* = 700 s/mm2) with acquisition parameters: 2.0 × 2.0 mm^2^ in-plane resolution, 2.0 mm slice thickness, FOV = 256 × 256 mm2, matrix = 128 × 128, TE = 84 ms, and TR= 8.04 s.

### 2.5 Diffusion Data Processing

All diffusion data were pre-processed by DTIPrep for quality control followed by TRACULA (TRActs Constrained by UnderLying Anatomy) (Yendiki et al., 2011). Images in each diffusion weighted imaging (DWI) series were aligned to the first non-diffusion-weighted image using affine registration (Jenkinson and Smith, 2001), (Leemans and Jones, 2009; Rohde et al., 2004). The TRACULA-outputted fractional anisotropy (FA) maps were further processed by Track-Based Spatial Statistics (TBSS) (Smith et al., 2004; Smith et al., 2006) The FA volumes were non-linearly aligned to a common space. FMRIB58_FA image was used as the target image for a linear registration to the standard space. Each participant's mean diffusion measure image was generated and thinned to create an alignment-invariant tract representation (the ‘skeleton') representing the centers of all tracts common to the group. The group data were thresholded at 0.2 before statistical testing.

### 2.6 Whole-Brain Diffusion Analysis

Voxelwise analyses on the FA, mean diffusivity (MD), axial diffusivity (AD), and radial diffusivity (RD) were carried out in Tract-Based Spatial Statistics (TBSS) (Smith et al., 2004; Smith et al., 2006) using general linear models by regressing the CBCL-ED score against each diffusion measure throughout the whole brain to identify significant regions correlated with ED symptom severity. Non-parametric randomized permutation test was performed (number of permutations = 5000) (Winkler et al., 2014), correcting for multiple comparisons using the threshold-free cluster enhancement method (Smith and Nichols, 2009) and controlling for family-wise error rate with a threshold of *p* < 0.05. For any TBSS-significant findings, individual mean diffusion weighted imaging (DWI) values were averaged across significant voxels in the standard space for visualization in the scatter plot.

### 2.7 Quality Assurance

Four DTI motion measures were derived by TRACULA (Yendiki, 2014), including the average translation score, rotation score, signal drop-out score (percentage of bad slices), and signal drop-out severity (Yendiki, 2014; Benner et al., 2011). A composite head motion score was computed for each participant based on these 4 motion measures (Yendiki, 2014). In all analyses, the individual motion composite score, and age, data source, lifetime history of mood disorders were modeled as nuisance covariates controlling for the impacts from these factors.

### 2.8 Tract-Based Region-Of-Interest (ROI) Analysis

To determine specific locations within tracts of interest associated with ED based on the whole-brain analyses, tract-based analyses were performed on the TRACULA outputted DWI values along each ROI tract's ‘spline representation’ (the average center location of the tract). Individual DWI values along each tract were interpolated from the native space to standardized positions for group statistical tests on comparable spline location points. For each spline point along each tract of interest, Spearman's correlation test was applied across group to determine the relationship between ED symptom severity and DWI measures scores for that location. P value < 0.05 was applied to threshold the results. Locations larger than three consecutive points passing the threshold were considered significant.

## 3 Results

### 3.1 Whole-brain TBSS Results

**Fig. 3:**
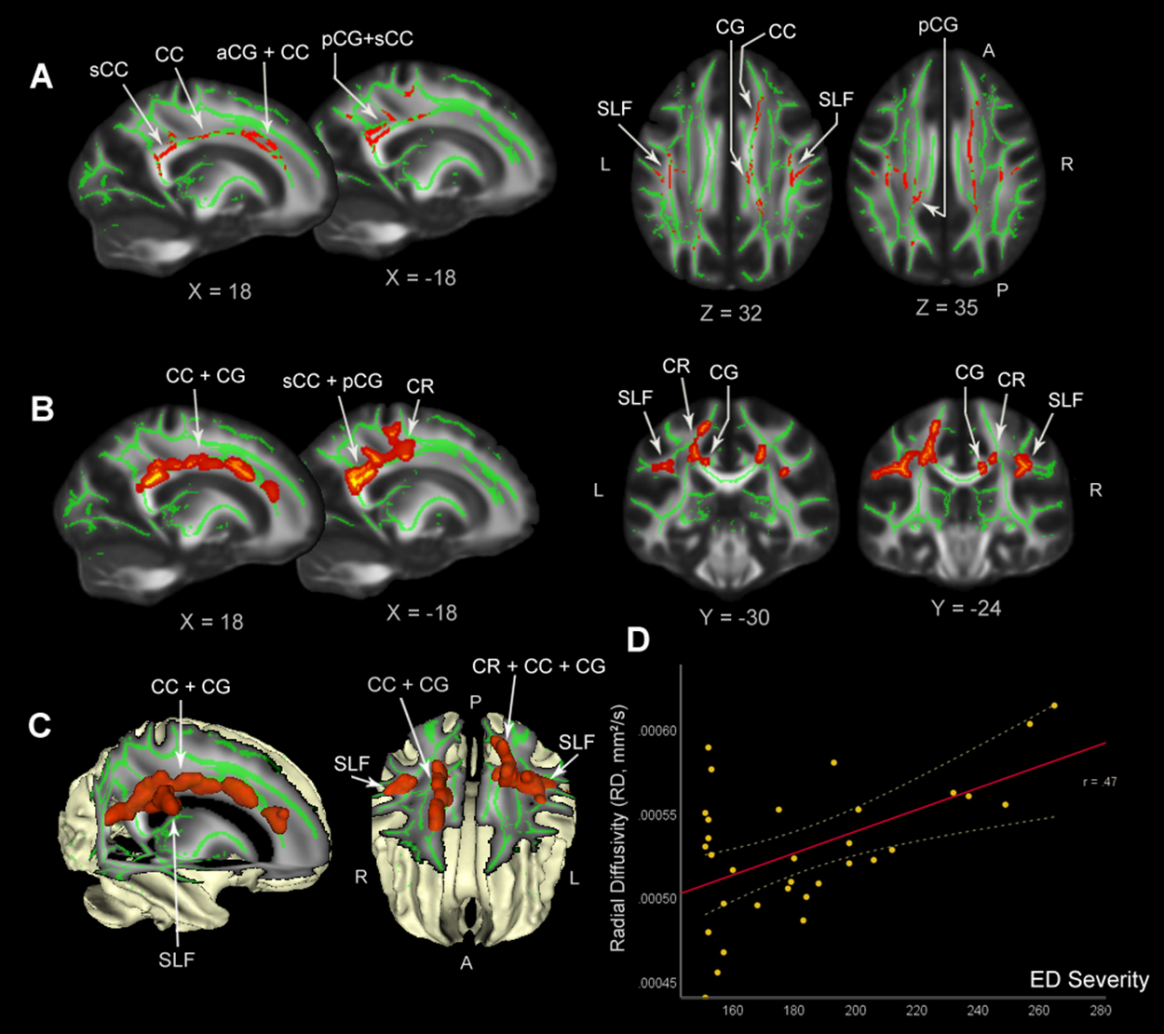
TBSS whole-brain results of significant white matter regions positively correlated with emotional dysregulation characterized by CBCL-ED. (3A) The skeleton map red color shows where RD significantly and positively correlated with ED symptom severity along the cingulum (CG) and corpus callosum (CC), specifically including the anterior and posterior subdivisions of the cingulum (aCG; pCG) that are connected with the body and the splenium of corpus callosum (CC; sCC) and the anterior to superior corona radiata (CR), extending laterally into small clusters of the SLF. (3B) The significant brain areas are filled into the mean FA map to visualize implicated local tracts (red color). (3C) 3D rendering of the TBSS-significant filled images in standard template brain, where RD significantly showed a positive relationship with the severity of ED symptoms. All images are threshold with *P* < 0.05 voxelwise and corrected by multiple comparisons. The FMRIB58 1 mm standard FA template image is used for overlay display. The JHU DTI-based atlases are used (https://fsl.fmrib.ox.ac.uk/fsl/fslwiki/Atlases) to determine white-matter locations of significant results. (3D) Scatter plot visualizes mean RD values extracted from the significant brain regions (Y-axis) plotted against the CBCL-ED scores (X-axis, with 150 being the lowest obtainable ED score) and with the prediction line (middle line) and 95% confidence interval (curved lines). The plot shows that worse (higher) CBCL-ED scores are associated with higher values of RD. The Spearman's correlation coefficient of the relationship (rs) is displayed for illustrational purposes.

TBSS voxel-wise analysis of all 32 children showed a significant positive correlation between RD and CBCL-ED score (*P* < 0.05 voxel-wise, corrected for multiple comparisons) located in the cingulum (CG) and corpus callosum (CC) pathways (Fig. 3A - 3C), including the anterior and posterior subdivisions of the cingulum (aCG; pCG), connected with the body and the splenium of CC and (the anterior and superior) corona radiata (CR), and extending laterally into small clusters of the superior longitudinal fasciculus (SLF). The individual RD extractions from the TBSS-significant regions visualized their significant positive relationship with the ED symptom severity (Fig. 3D; Spearman's Rho = 0.47, *P* = 0.002). No other significant results were found with FA, MD, or AD measures at the whole-brain level.

### 3.2 Tract-based Results

**Fig. 4:**
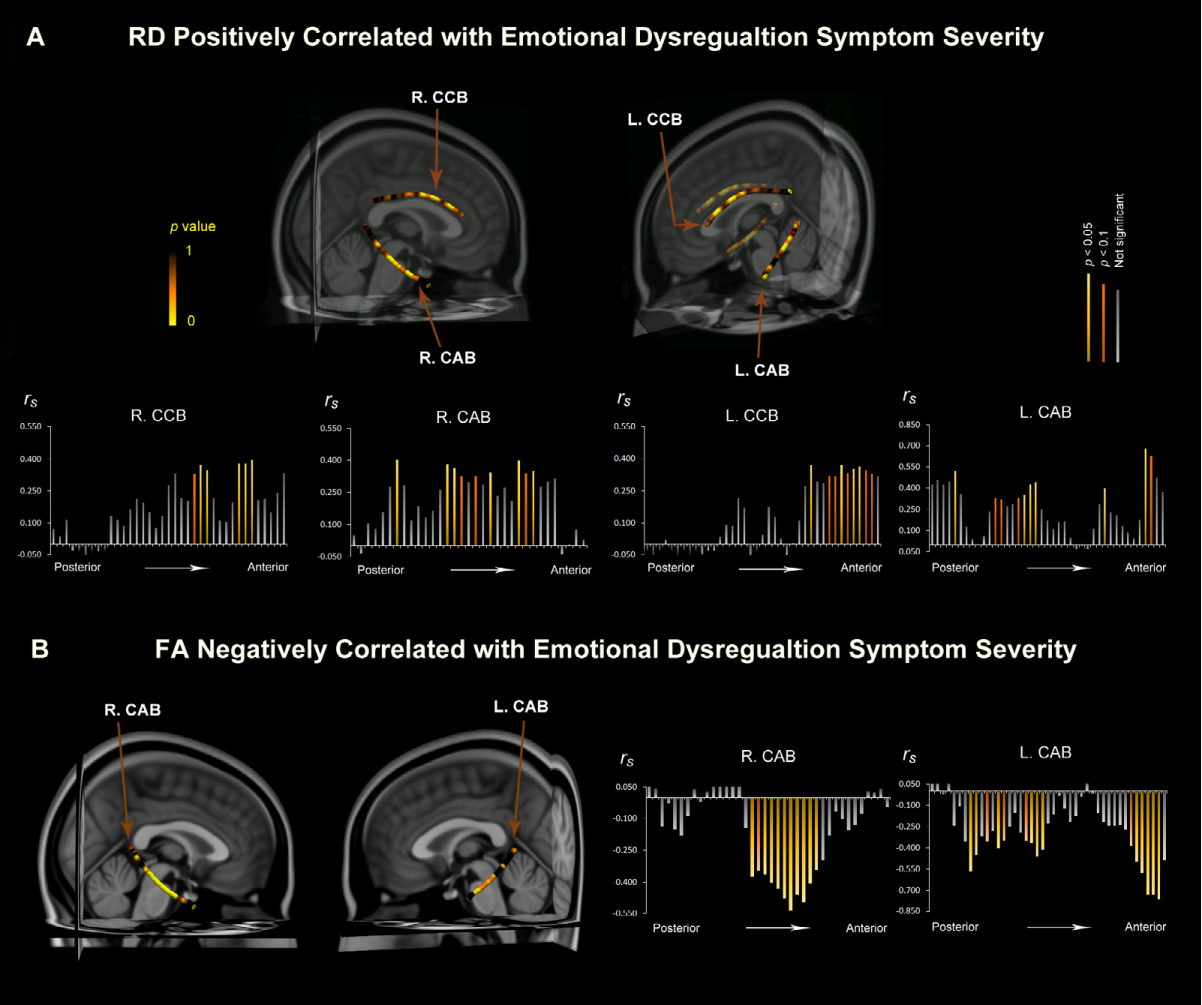
Tract-based results of significant regions where diffusivities significantly correlate with ED. Post-hoc examinations along tracts of interest show significant locations of positive correlations between RD and ED (yellow color in brain images in 4A) and negative correlations between FA and ED (4B) along the bilateral cingulum bundles (*P* < 0.05; dorsal bundle = cingulum cingulate bundle, CCB; ventral bundle = cingulum angular bundle, CAB). The bar graphs visualize the strength of the correlations (Y-axis, Spearmans’ correlation coefficient) along each tract's spline point (X-axis).

FA and RD measures were examined in relation to ED for tracts of interest as the post-hoc assessment on each spline point for the dorsal and ventral cingulum bundles bilaterally (dorsal bundle: cingulum cingulate bundle/CCB; ventral bundle: cingulum angular bundle/CAB), and the parietal and temporal bundles of the SLF bilaterally (parietal bundle: SLFp; temporal bundle: SLFt). Results of FA analysis showed significant locations of negative correlations with ED score along ventral bundles of cingulum bilaterally (CAB; *P* < 0.05, Spearman's correlation; Fig. 4B). Results of RD analysis showed significant positive correlations with ED along all bilateral cingulum bundles (CCB and CAB; *P* < 0.05, Spearman's correlation tests; Fig. 4A).

## 4 Discussion

Using whole-brain diffusion weighted imaging and a dimensional framework that aligns with the NIH Research Domain Criteria (Insel et al., 2010; Versace et al., 2015), this study revealed that pediatric emotional dysregulation (ED), as a clinical dimension independent from psychiatric diagnosis, is specifically linked to **variation** in white-matter microstructure within cingulum-callosal neurocircuitry. Children with greater ED severity showed increased RD and decreased FA in the cingulum-callosal regions. These results contribute to improved understanding of the etiological nature of pediatric mood disorders. These findings suggest that abnormal diffusivity of this circuitry may represent a developmental risk biomarker for bipolar mood disorders and other syndrome-congruent disabilities.

### 4.1 Myelination related susceptibility reflected by increased RD and decreased FA

In normal myelinated axons, diffusion is restricted in directions perpendicular to the axon as characterized by RD. Elevated RD indicates that the water molecules diffuse more freely and are less restricted in perpendicular directions in the axons. RD has been demonstrated to be particularly sensitive to demyelination, or changes in the axonal diameters or density, as reported in animal and ex-vivo studies (Alexander et al., 2007; Alexander et al., 2011; Feldman et al., 2010). Animal studies have suggested RD as an indicator of myelin damage (Aung et al., 2013) and RD has been observed to correlate with myelin degradation in mice (Song et al., 2003). Moreover, increased RD corresponded to demyelination in the corpus callosum, followed by normalization of RD in the recovery stage during remyelination (Song et al., 2005; Sun et al., 2006; Xie et al., 2010). Unlike RD, the FA measures the strength of directionality of the local white-matter tract. Decreases in FA coupled with increases in RD, further indicate potential underlying problems related to myelination (Song et al., 2002). Taken together, the current findings of increased emotional dysregulation severity associated with increased RD and decreased FA suggest possible neural susceptibility related to axonal myelination along the cingulum and callosal pathways, which contribute to emotional dysregulation in children.

### 4.2 Implicated cingulum-callosal neurocircuits underlying emotional dysregulation

We found that emotional dysregulation in children correlated with microstructures in the cingulum and connected callosal pathways that extend laterally into corona radiate. The cingulum and callosal bundles constitute the largest white matter tracts in the brain, connecting dorsal cortical brain areas (including the frontal, visuoparietal, and sensorimotor systems) with ventral brain areas in the medial temporal lobe, including the limbic system (Catani and Thiebaut de Schotten, 2012; Schmahmann and Pandya, 2009). These are neural pathways implicated in emotional attribution, attentional allocation, and executive control, all of which play key roles in emotion regulation (Buhle et al., 2014; Ochsner and Gross, 2005; Vandekerckhove et al., 2020). The cingulum bundle primarily receives neuronal inputs from the cingulate cortex (Catani and Thiebaut de Schotten, 2012), the brain region implicated in the regulation of cognitive and emotional control processes that is altered in mood disorders (Bush et al., 2000; Giuliani et al., 2011; Versace et al., 2015; Wessa and Linke, 2009). The callosal fibers connect the two hemispheres and extend into sensorimotor regions (via the corona radiata) and are involved in sensorimotor coordination and maintaining balance of arousal and attentional vigilance (Rueckert and Levy, 1996; Rueckert et al., 1999; Sauerwein and Lassonde, 1994).

The broad-range connections of cingulum and callosal pathways support the multi-faceted nature of the clinical phenotype of ED operationalized via empirically-derived CBCL-ED, including components of the Attention, Aggression, and Anxiety/Depression dimensions. The cingulum and callosal fibers integrate cortical structures including the cingulate, fronto-parietal regions, and temporoparietal junction, which together constitute large-scale executive, attentional, and motivational neurocircuitries connected by long-range white matter tracts allowing for network synchronization (Catani and Thiebaut de Schotten, 2012). Imbalance between the dorsal brain network (including the anterior and posterior cingulate and prefrontal cortical regions) and the ventral brain network (including the limbic system) has been proposed to underlie deficient emotion regulation in mood disorders, particularly bipolar disorder (Wessa and Linke, 2009). This imbalance involves not only compromised down-regulation of the emotion-related ventral, limbic activity, but also insufficient up-regulation of the excitatory dorsal, frontal activity.

The current pediatric finding complements previous evidence for associations between cingulum differences and emotional dysregulation in adolescents and adults. The correlation between diffusion from the cingulum region and the emotional dysregulation severity measured by the CBCL-ED tool is consistent with a region-of-interest spectroscopy study (Wozniak et al., 2012) that examined the relationship between the anterior cingulate cortex glutamate concentrations levels and severity of ED measured by the CBCL-ED scores. Increases in ACC glutamate levels were positively related to ED severity among participants with abnormal ED profiles (CBCL-ED scores > 180) (Wozniak et al., 2012). In addition, greater cingulum fiber length predicted lower mania scores in adolescents with ED symptoms compared with controls (Bertocci et al., 2016). Increased AD was associated with more severe manic symptoms in the cingulum tract in emotionally dysregulated adolescents compared with controls (Versace et al., 2015). Higher FA in the cingulum was associated with high emotional approach, and increases in MD in the body and the splenium of the corpus callosum were associated with low emotional approach in healthy adult females (Vandekerckhove et al., 2020). Furthermore, corpus callosum fibers were also found to be thinner in adult patients with bipolar disorder relative to healthy controls, with particularly reduced thickness in the splenium (Walterfang et al., 2009).

### 4.3 Clinical implications: risk neuromarker for pediatric mood disorders

Clinical evidence suggests that the CBCL-ED profile (or the CBCL A-A-A score) is useful in the differential diagnosis of pediatric mood disorders and in the longitudinal prediction of mood disorders. Clinical data has shown that the CBCL-ED score effectively assisted with diagnostic precision in structured interviews to sensitively identify mood disorders in youth (Carlson and Kelly, 1998; Faraone et al., 2005; Geller et al., 1998; Hazell et al., 1999; Mick et al., 2003; Uchida et al., 2014; Wals et al., 2001), and has been associated with current and future diagnosis of mood disorders (Spencer et al., 2011; Uchida et al., 2014). An elevated CBCL-ED score (i.e., A-A-A score > 180) has identified children with increased susceptibility to developing mood disorders, and a severely elevated ED profile (i.e., A-A-A score ≥ 210) was particularly sensitive for identifying children who were later diagnosed with pediatric bipolar disorder (Arnold et al., 2011; Biederman et al., 2012a; Biederman et al., 2012b; O'Brien and Frick, 1996). Further, the CBCL-ED score was predictive of children developing mood disorders in a 10-year prospective longitudinal study: A high CBCL-ED score at baseline predicted subsequent diagnoses of major depressive disorder and bipolar disorder, as well as syndrome-congruent functional impairments ranging from school problems to interpersonal difficulties and higher risk for psychiatric hospitalization (Biederman et al., 2009).

In the current study, we found that the CBCL-ED score, which is clinically associated with risks for development of mood disorders in general and bipolar disorder in particular, is linked with compromised microstructure along the cingulum-callosal brain connection tracts, implicating possible etiological changes that are frequently reported in mood disorders (Bush et al., 2000; Giuliani et al., 2011; Versace et al., 2015; Wessa and Linke, 2009). Converging evidence has indicated that abnormalities in the frontotemporal white-matter development are highly implicated in the emotional dysregulation characteristic in bipolar mood disorders (de Zwarte et al., 2014). Impaired white-matter integrity in children and adolescents with bipolar illness has been found in the pericingulate and mid-posterior cingulate-callosal fibers extending into the parietal-occipital corona radiata regions (Barnea-Goraly et al., 2009; de Zwarte et al., 2014; Frazier et al., 2007; Gao et al., 2013; Gonenc et al., 2010) as well as observed in the callosal regions (Caetano et al., 2008). The cingulum-callosal bundles connect the dorsal cortical brain systems with the limbic system, particularly via its posterior-to-inferior (angular) bundle. These brain circuits connect the cortico-limbic emotional system, the fronto-parietal attentional system, and the frontal motor-control system. The CBCL-ED measurement may index this brain network because the A-A-A profile captures the neuropsychological constructs (Anxiety/Depression, Attention, and the Aggression) supported by cingulum-callosal structures linking the required brain networks.

### 4.4 Limitations and future directions

The current DTI measure did not fully characterize crossing fibers, thus we are limited to interpreting the small significant regions of the SLF that could intersect with corona radiata and cortical spinal tract. Future research using more advanced microstructural measures (e.g., diffusion spectrum imaging) may further characterize potential attribution of brain regions of emotional dysregulation in developmental cohorts. Incipient pediatric mood disorders can follow a unipolar or bipolar course, which require different treatment approaches. This prompts future work aimed at identifying clinical biomarkers of risk to help identify children at risk of one type of mood disorder or another.

## 5 Conclusions

The present study provides the first whole-brain neurobiological account underlying the pathophysiological risk mechanism of ED for pediatric mood disorders. Findings revealed that weakened microstructural integrity in the cingulum-callosal pathways is associated with elevated severity of ED symptoms. Altered cingulum and callosal microstructure may manifest as a susceptibility neural biomarker predictive of a potential pathological course towards mood disorders. New insights from this study may shed light on ongoing efforts to find sensitive predictive tools, develop preventive intervention strategies, and improve treatment precision and outcomes.

## Funding

This study was supported by the DuPont Warren Fellowship Award, the Livingston Award, the Pediatric Psychopharmacology Council Fund at the Massachusetts General Hospital, and the Poitras Center for Psychiatric Disorders Research at the McGovern Institute for Brain Research at Massachusetts Institute of Technology. This work was also supported by the Brain and Behavior Research Foundation’s NARSAD Young Investigator Award and Canadian Institutes of Health Research Fellowship Award. Authors SG and MG were partially supported by NIH grants R01 EB020740, P41 EB019936, and U01 MH108168. The funding sources were not involved in the study design; in the collection, analysis, and interpretation of data; in the writing of the report; and in the decision to submit the paper for publication.

## Author contributions

dr.. Yuwen Hung and dr. Mai Uchida had full access to all the data in the study and take responsibility for the integrity of the data and the accuracy of the data analysis. Conception of Design: dr. Yuwen Hung, dr. Mai Uchida; Acquisition, Analysis, or Interpretation of Data: Schuyler Gaillard, James Capella, Hilary Woodworth, Caroline Kelberman, Kelly Kadlec, Mathias Goncalves, dr. Satrajit Ghosh, dr. Anastasia Yendiki, dr. Susan Whitfield-Gabrieli, dr. John Gabrieli and dr. Joseph Biederman; Drafting of the Manuscript: dr. Yuwen Hung and dr. Mai Uchida; Critical Revision of the Manuscript for Important Intellectual Content: Schuyler Gaillard, James Capella, Hilary Woodworth, Caroline Kelberman, Kelly Kadlec, Mathias Goncalves, dr. Satrajit Ghosh, dr. Anastasia Yendiki, dr. Susan Whitfield-Gabrieli, dr. John Gabrieli, and dr. Joseph Biederman; Statistical Analysis: dr. Yuwen Hung and dr. Mai Uchida; Obtaining Funding: dr. Mai Uchida; Administrative, Technical, or Material Support: Schuyler Gaillard, James Capella, Hilary Woodworth, Caroline Kelberman, Kelly Kadlec, Mathias Goncalves, dr. Satrajit Ghosh, dr. Anastasia Yendiki and dr. Susan Whitfield-Gabrieli; Supervision: dr. John Gabrieli and dr. Joseph Biederman.

## Appendix Supplementary materials

Image, application 1
