# Supplementary material for: Cingulum-Callosal white-matter microstructure associated with emotional dysregulation in children: A diffusion tensor imaging study
Source: Neuroimage Clin. 2020 Apr 25;27:102266. doi: 10.1016/j.nicl.2020.102266 (PMC7218214; doi:10.1016/j.nicl.2020.102266)
Supplement: Supplementary file 1 [file mmc1.docx]

**Supplementary Materials**

**
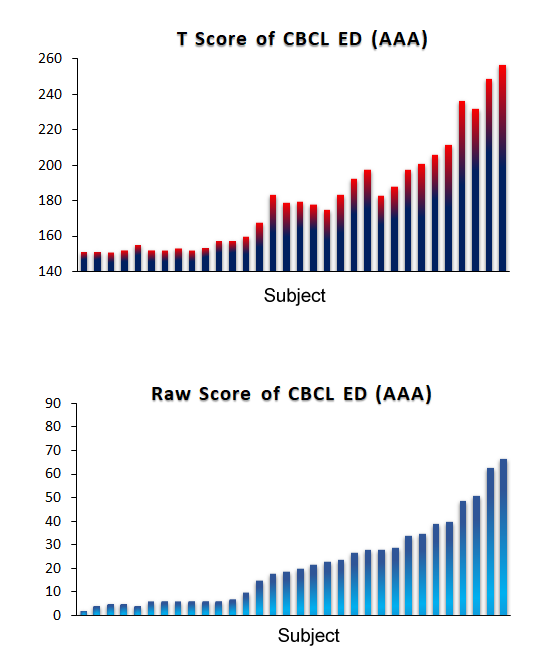
**

**Supplementary Figure 1.** The CBCL-ED T score and raw score distributions.

The top figure shows the T scores of CBCL-ED and the bottom figure shows the raw scores of CBCL-ED for all study participants. The CBCL-ED score (or the CBCL A-A-A score) is the total score combining the three CBCL syndrome scales: The Anxious/Depressed, the Attention Problems, and the Aggressive Behavior subscales.
